# Advancements in Cytomegalovirus Management Among Solid Organ Transplant Recipients: Insights From the ESOT CMV Workshop 2023

**DOI:** 10.3389/ti.2025.14195

**Published:** 2025-08-22

**Authors:** Luca Toti, Nassim Kamar, Sophie Alain, Oriol Manuel, Nikolina Basic-Jukic, Paolo Antonio Grossi, Hannah Kaminski, Paolo Solidoro, Luciano Potena

**Affiliations:** ^1^ Department of Surgical Sciences, University of Rome “Tor Vergata”, Rome, Italy; ^2^ Department of Nephrology and Organ Transplantation, Toulouse Rangueil University Hospital, INFINITY-Inserm U1291-CNRS U5051, University Paul Sabatier, Toulouse, France; ^3^ Department of Virology and National Reference Center for Herpesviruses, Limoges University Hospital, UMR Inserm 1092, University of Limoges, Limoges, France; ^4^ University Hospital Federation SUPORT, Limoges, France; ^5^ Infectious Diseases Service and Transplantation Center, University Hospital and University of Lausanne, Lausanne, Switzerland; ^6^ Department of Nephrology, Arterial Hypertension, Dialysis and Transplantation, University Hospital Centre Zagreb, School of Medicine University of Zagreb, Zagreb, Croatia; ^7^ Università degli Studi dell’Insubria - ASST SetteLaghi, Varese, Italy; ^8^ Department of Nephrology, Transplantation, Dialysis and Apheresis, Centre Hospitalier Universitaire Bordeaux, Bordeaux, France; ^9^ S.C.U Pneumologia, Azienda Ospedaliero Universitaria Città della Salute e della Scienza, Department of Medical Sciences, University of Turin, Turin, Italy; ^10^ Unit for Heart Failure and Transplantation, IRCCS Azienda Ospedaliero-Universitaria di Bologna, Bologna, Italy

**Keywords:** cytomegalovirus, solid organ transplant recipients, universal prophylaxis, pre-emptive therapy, cell-mediated immunity

## Abstract

Cytomegalovirus (CMV) infection poses significant challenges in solid organ transplant (SOT) recipients, impacting graft outcomes, morbidity, and in some cases survival. The ESOT CMV Workshop 2023 convened European experts to discuss current practices and advances in the management of CMV with the aim of improving the quality of life of transplant recipients. Discussions covered crucial areas such as preventive strategies, diagnostic challenges, therapeutic approaches, and the role of cell-mediated immunity (CMI) monitoring. Despite advances, ambiguity persists in optimal CMV management across European transplant centers. Preventive strategies, including universal prophylaxis and pre-emptive therapy, are effective but consensus is lacking with respect to the preferred approach. Diagnostic challenges such as standardization of viral load thresholds and detection of end-organ disease complicate timely intervention. While newer therapies like maribavir hold promise for treating complicated CMV infections, sustaining viral clearance remains a challenge. Integrating CMI monitoring into CMV management could personalize treatment decisions but has limitations in in terms of predictive value and accessibility. Further research is needed to fill these gaps and optimize CMV management. The collaborative efforts, led by the European Society for Organ Transplantation (ESOT), aim to standardize and improve CMV care, ensuring better outcomes for SOT recipients.

## Introduction

Cytomegalovirus (CMV) is a widespread herpes virus [1]. CMV seroprevalence affects approximately 80% of the global population, and tends to increase with age [2]. However, there is considerable inter-country variation. France has reported a CMV seroprevalence point estimate of 41.9% among individuals aged 15–49 years, whereas Croatia has reported an overall rate of 74.4% [3, 4]. Although CMV infection is usually asymptomatic or results in only mild disease in the general population, it can lead to severe outcomes in patients who are immunosuppressed, particularly solid organ transplant (SOT) recipients, where latent CMV infection may reactivate and lead to CMV disease [1, 5]. Post-transplant CMV disease may also result from transmission through an infected transplanted organ in seronegative patients [1, 5], significantly impacting graft loss, morbidity and occasionally mortality [6–8]. CMV disease typically occurs within 3 months of transplant (early-onset), although onset may be delayed when antiviral prophylaxis is preferred (late-onset) [5, 9]. Invasive disease can result as a direct cytopathic effect of CMV in organs, manifesting as pneumonia, gastrointestinal (GI) tract disease, hepatitis, encephalitis, and retinitis. CMV infection can also indirectly impact graft function and exacerbate the risk of opportunistic infections [5].

The management of CMV disease in SOT recipients varies considerably across different European centers, highlighting the absence of standardized care protocols [10, 11]. The European Society for Organ Transplantation (ESOT) organised a 1-day workshop on the “Management of CMV in solid organ transplant recipients” in Milan, Italy, on 17 November 2023 with the primary objective of discussing strategies to harmonize CMV management practices across Europe. Experts in the field discussed historic and current diagnostic and therapeutic approaches to the management of CMV. The workshop provided an opportunity for delegates involved in CMV management to share country-specific insights and explore strategies aimed at improving treatment outcomes in SOT settings. Consisting of five expert-led sessions covering CMV prevention, testing, diagnosis, management, and immune monitoring, and complemented by interactive case study sessions, the workshop aimed to elucidate key insights and strategies for improving treatment outcomes in SOT settings. This meeting report summarizes clinical cases analyzed during the workshop, focusing on opportunities to improve outcomes for transplant recipients through cell-mediated immunity (CMI) and the management of resistant or difficult-to-treat CMV disease and reviews the highlights and emerging trends discussed during the workshop, offering valuable insights into the evolving landscape of CMV management in SOT recipients. The Scientific Leads determined the three topics for the case studies: CMV disease, CMV resistance, and immune monitoring for CMV. The three case studies were then independently developed by the faculty.

## Management of CMV Infection and Disease

### The Relationship Between CMV and Patient Outcomes

CMV is the most common pathogen detected after SOT and is associated with significant morbidity and in some cases may lead to death or graft loss [12]. Therefore, understanding the relationship between CMV and patient outcomes post-transplantation is critical. CMV infection has complicated SOT since the first procedure [13]. In a 1964 study by Hill RB et al, among the 61 SOT recipients included, 32 died (mainly kidney recipients), with a median survival post-transplant of only 36.5 days. Notably, autopsy findings revealed that 26 of these patients had active pulmonary infection, with CMV identified as the predominant pathogen in 58% of cases, suggesting a possible association between CMV pneumonitis and mortality [14]. In addition to direct effects related to organ-specific infections, later reports showed an association between CMV infection and acute or chronic graft rejection. In a pivotal study from Grattan MT et al, CMV infection was found to be associated with acute rejection and coronary artery disease in heart transplant recipients [15]. More recently, in a retrospective cohort study involving 192 kidney transplant recipients, patients with CMV disease had a significant likelihood of developing acute rejection after CMV infection or reactivation [16]. Additionally, in 2014 Stern and colleagues conducted a study involving 1414 recipients of heart, kidney, liver, or lung allografts, revealing an increased risk of biopsy-proven graft rejection within 4 weeks after CMV replication was detected [17].

Advances in screening, prophylactic antiviral therapy, and pre-emptive treatment have mitigated the impact of CMV disease on morbidity and mortality following SOT. However, although significant improvements have been made, in the current era, morbidity and mortality data related to CMV disease during organ transplantation remain variable despite advancements in antiviral treatments and the use of newer immunosuppressive drugs [18].

**FIGURE 1:**
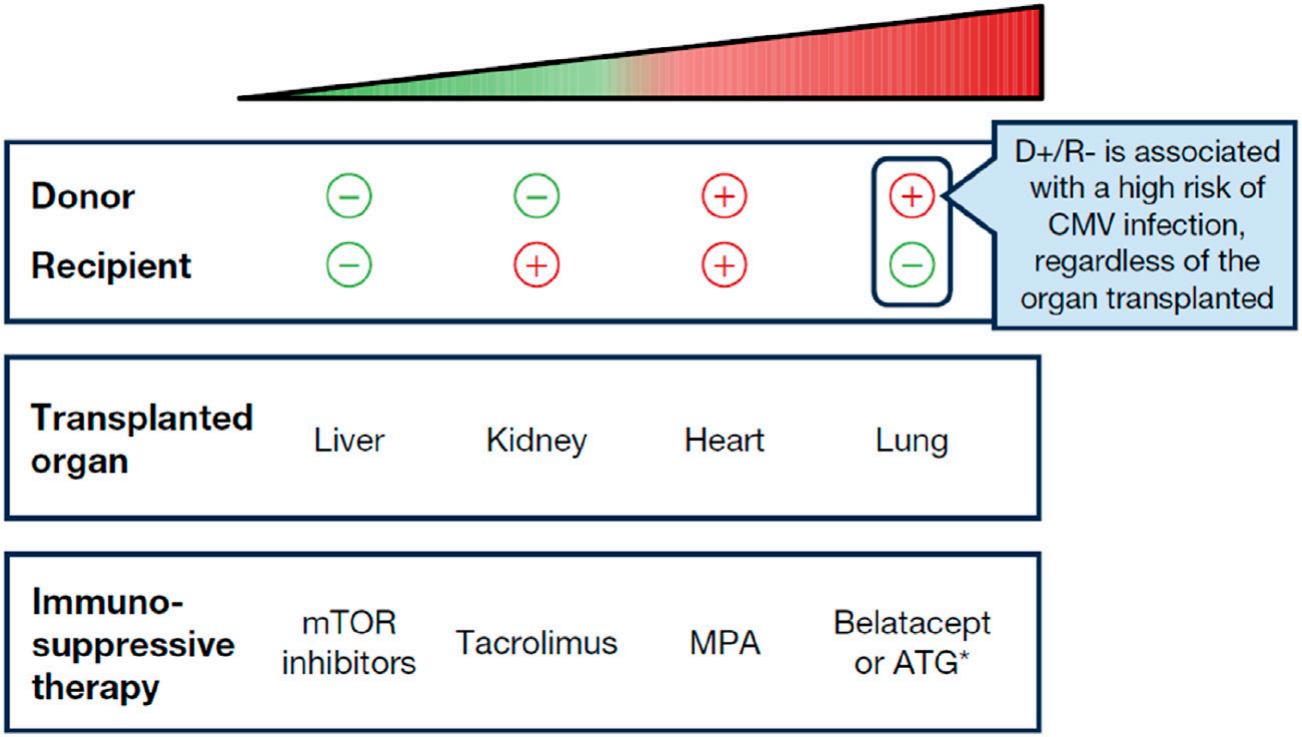
The “hierarchy” of risk with respect to CMV infection, from lowest to highest risk. *Only a higher risk in R+ patients. The choice of immunosuppressant therapy may vary depending on the organ transplanted as certain immunosuppressive therapies may not be suitable for all types of transplants. MPA, mycophenolic acid; mTOR, mammalian target of rapamycin.

**FIGURE 2:**
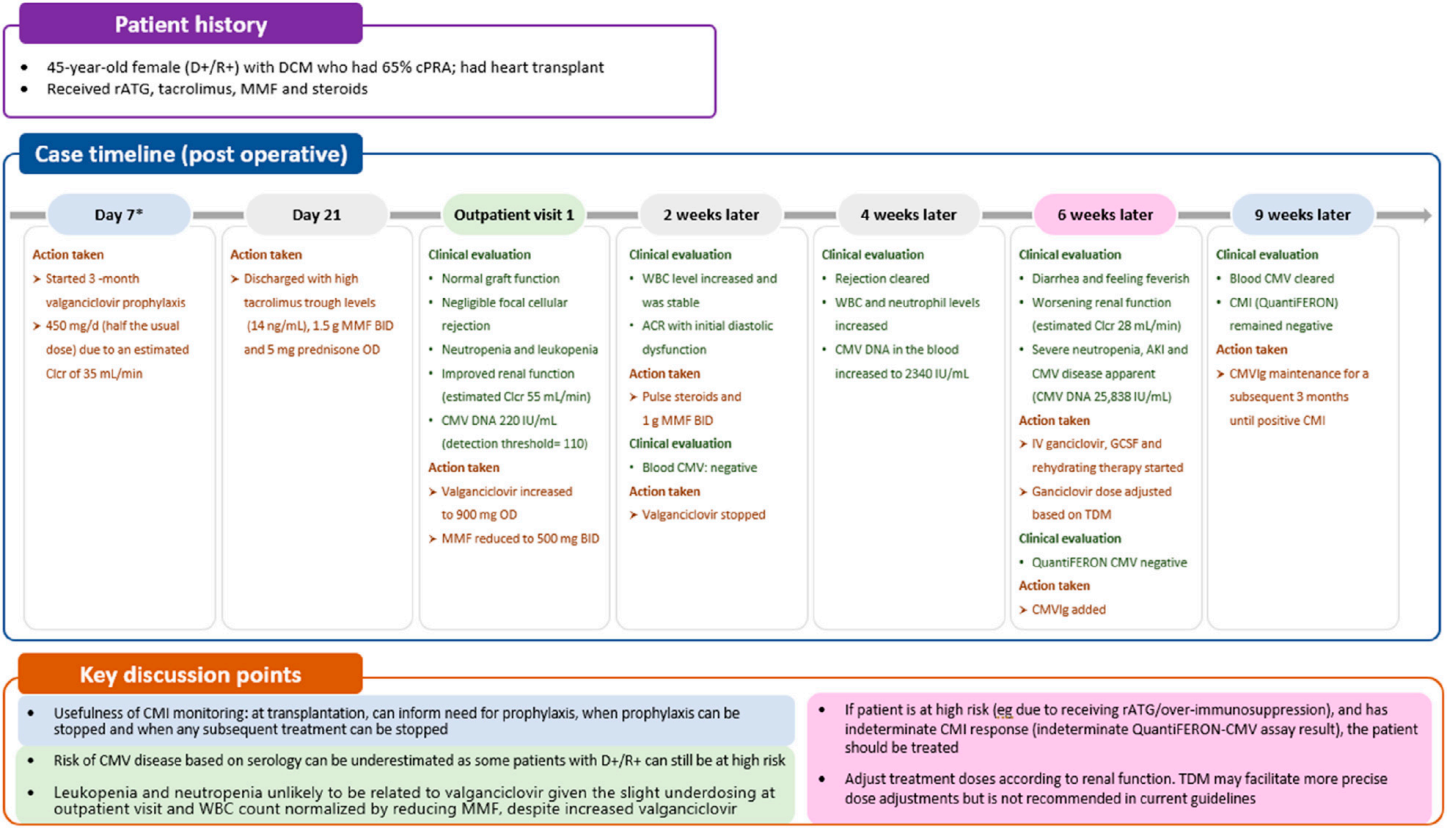
Case 1: immune monitoring for CMV. *First available evaluation. ACR, acute cellular rejection; AKI, acute kidney injury; BID, twice daily; Clcr, creatinine clearance rate; CMI, cell-mediated immunity; CMV, cytomegalovirus; CMVIg, cytomegalovirus immunoglobulin; cPRA, calculated panel reactive antibody; DCM, dilated cardiomyopathy; GCSF, granulocyte colony-stimulating factor; IV, intravenous; MMF, mycophenolate mofetil; OD, once daily; rATG, rabbit antithymocyte globulin; TDM, therapeutic drug monitoring; WBC, white blood cell.

Likelihood of CMV infection in patients undergoing SOT is influenced by several factors. The most significant risk factor is the serological status of the donor (D) and recipient (R), determined by the presence or absence of anti-CMV antibodies. The highest risk of CMV infection occurs when an organ from a CMV-seropositive donor (D+) is transplanted into a CMV-seronegative recipient (R-), designated (D+/R-). Consequently, pre-transplant screening is widely acknowledged to be of paramount importance [19]. Additionally, the type of organ transplanted also affects the CMV risk, with lung transplant recipients facing the highest susceptibility, followed by heart, kidney, and liver recipients (Figure 1). Thus, the riskiest scenario regarding CMV infection involves lung transplantation from a seropositive donor to a seronegative recipient (D+/R-). The level of immunosuppression is also important to consider, with the administration of lymphocyte-depleting antibodies (i.e., anti-thymocyte globulin [ATG]) as induction and/or anti-rejection therapy also being associated with increased incidence of CMV, in seropositive recipients. Of note, risk stratification based on donor and recipient serology may only partially estimate the risk for CMV disease. Case 1 underscores the importance of considering all the factors associated with CMV infection (Figure 2), such as in a D+/R+ scenario in the presence of additional risk factors like the need for increased immunosuppression.

### Strategies to Improve CMV Infection and Disease Outcomes

**FIGURE 3:**
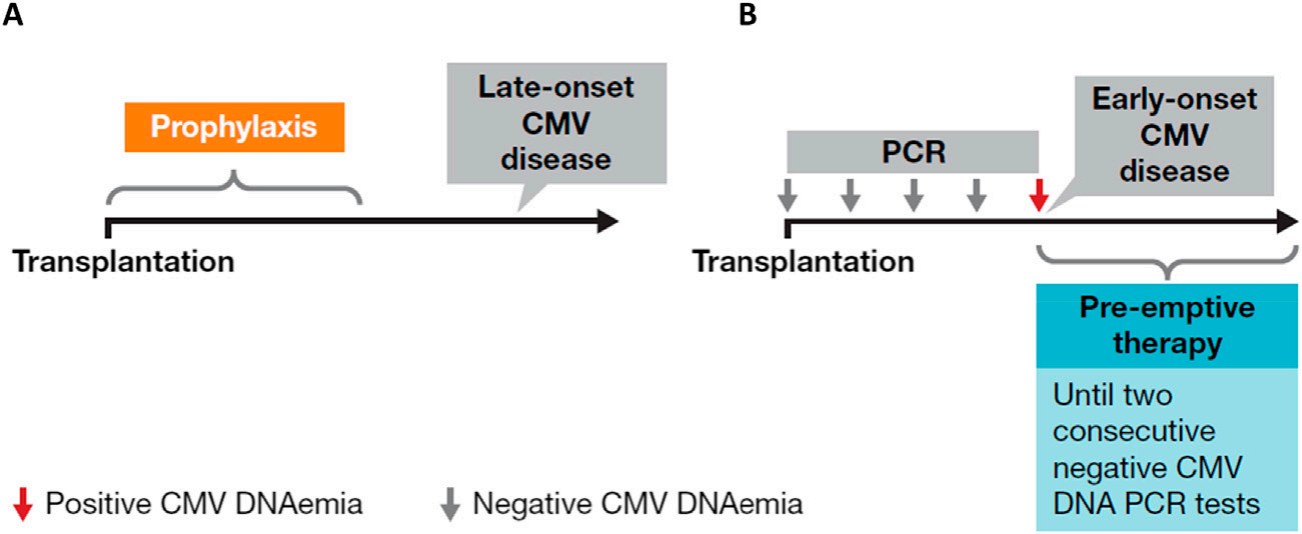
Two available preventive strategies for CMV: **(A)** universal prophylaxis and **(B)** pre-emptive approach. Figure was created by Oriol Manuel. Adapted and reproduced with permission from [20].

Given the profound implications of CMV disease in SOT recipients, effective CMV prevention strategies can enhance the success and improve the outcomes of transplant procedures. Two preventive strategies are available (Figure 3): universal prophylaxis (administration of antivirals to all patients at risk, starting within 10 days after transplant and continuing for at least 3 months and up to 12 months in D+/R- lung transplant recipients [duration dependent on organ transplanted and D/R serostatus]) or pre-emptive therapy (monitoring for DNAemia every week, followed by the administration of antivirals until at least two consecutive negative DNAemia tests are achieved or according to center-specific thresholds) [6]. Both strategies are effective in preventing CMV disease, with no consensus on the superiority of one over the other, but with prophylaxis preferred in lung transplants and pre-emptive therapy in liver [21–23]. The choice between universal prophylaxis and pre-emptive therapy will be driven by the expected relative benefits of each strategy (Table 1) as well as the clinical situation in the individual patient. As an illustration, in case 1, a patient (D+/R+) undergoing a heart transplant, who based on serology and transplanted organ could be considered to be at lower risk of CMV infection than a seronegative recipient and a candidate for pre-emptive therapy, received universal prophylaxis with valganciclovir due to the increased risk of CMV disease associated with potent immunosuppression (Table 1; Figure 2).

**TABLE 1:** An overview of authors’ consensus on preventive measures for CMV.

Preventive strategy	Universal prophylaxis	Pre-emptive approach
Criteria	• Given to all patients at risk	• Weekly monitoring of viremia during the first 3 months and then twice a month from months 3–6 • Antivirals continued until at least two consecutive negative DNAemia tests are achieved or according to center-specific thresholds^a^
Frequent preferred indications	• High-risk serostatus (D+/R-)^b^ • Lung transplant recipients • Potent immunosuppression (such as the use of belatacept or induction with ATG in R+ patients) • History of CMV reactivation • Limited monitoring capabilities • Individual patient factors	• Lower risk patients (CMV seropositive recipients and those not receiving ATG) [24] • May be given to D+/R- patients only in centres capable of performing strict and reliable monitoring of DNAemia^c^
Benefits [25]	• Easy to implement • Potential to prevent other herpes viruses (in the case of valganciclovir), other opportunistic infections and rejection • May influence graft function and may reduce the impact of indirect effects of CMV • Prevention of severe CMV disease • May be more appropriate in resource-limited settings where close monitoring is unavailable	• Reduced drug exposure • Preservation of immune response • Lower overall drug cost • Targeted treatment with individualized approach • Early detection of CMV reactivation • Patient-centered care
Challenges	• Associated with a higher incidence of late-onset CMV, so needs clinical monitoring after discontinuation of antivirals • High costs (for letermovir) • Drug toxicity (for valganciclovir) • Higher risk of antiviral resistance • Reduces immunobiological control • Difficulty in determining optimal duration of prophylaxis • Risk of drug interactions with other medications/immunosuppressives	• Requires close monitoring (risk of missed reactivation events) • No universal value for the initiation of treatment and cut-off values are center specific • Risk of over-treatment or under-treatment • Impact on indirect effects of CMV unknown • Does not address other herpes viruses • Higher logistic costs • Patient education and engagement

^a^ There is no consensus on a specific threshold but rather on a significant increase of viral load.

^b^ Preference is based on opinions at the workshop and is in line with the results of a survey conducted by ESOT in 2022 (in which 90% of respondents reported use of prophylaxis in D+/R- patients) [26] and current guidelines that support prophylaxis in kidney and cardiothoracic patients [19]. The situation is different for patients undergoing liver transplantation.

^c^ Grossi PA, et al. Transpl Int. 2022;35:10332 [26].

Valganciclovir is the standard of care for CMV prophylaxis in the most at-risk donor−recipient category (D+/R-) [27]. However, prolonged exposure in the setting of universal prophylaxis can lead to drug toxicity, in particular leukopenia [27]. A recent study by Limaye and colleagues demonstrated that letermovir is non-inferior in effectiveness to valganciclovir for CMV prophylaxis in D+/R- kidney transplant recipients, but with lower rates of leukopenia or neutropenia, suggesting its potential as a preferred option in D+/R- kidney transplant patients [27]. Furthermore, the use of CMV immunoglobulin (CMVIg) in combination with antivirals in CMV prophylaxis may be beneficial in specific circumstances, especially in D+/R- recipients of thoracic organs [28–30]. A meta-analysis assessed CMV infection rates in SOT patients who received prophylactic CMVIg, revealing a lower incidence of CMV infection in this cohort. Specifically, the average CMV infection rate was 35.8% (95% CI: 33.4%–38.2%) among patients who received CMVIg, compared with 41.4% (95% CI: 38.6%–44.2%) in the control group not receiving CMVIg (p = 0.003) [28]. Despite these promising results, the use of CMVIg remains controversial due to the lack of recent interventional data on efficacy.

### Challenges With Testing and Diagnosis Techniques for CMV and Strategies for Improvement

Unlike universal prophylaxis, a pre-emptive approach to CMV prevention requires regular monitoring for CMV viremia [6]. The quantitative nucleic acid amplification test detects and quantifies CMV DNA and is preferred over antigenemia. It has become the standard of care for diagnosing and monitoring post-transplant CMV infection [31]. Post-transplant monitoring typically occurs weekly during the first 3 months and then twice a month from months 3–6 as patients stabilize. Despite the international standardization of reporting all viral load values in IU/mL during the QNAT, a consensus around viral load thresholds remains a challenge because laboratory assays and matrix choices differ between centers. This leaves individual centers with the task of determining specific thresholds in their laboratories [26].

Studies have shown that an increase in viral load correlates with the occurrence of CMV disease [5]. Therefore, focusing on viral load trends over time is more useful and important for predicting disease development and guiding therapeutic decisions than using center-specific absolute viral load values, which lack standardization [5]. To illustrate this, in case 1, CMV DNA in the blood increased 10-fold in 2 weeks, which prompted treatment with an antiviral, granulocyte-colony stimulating factor (GCSF) and CMVIg. It is important to underline that DNAemia values from plasma and whole blood are not comparable [5], so it is crucial not to change tests or matrix choices during treatment and follow-up. Additionally, in our opinion, distinguishing the clinical significance of a viral load increase is complicated by free DNA release into plasma from infected cells, potentially leading to low-level or persistent DNAemia, which could be mistaken as an indication of active replication. In this context, assessment of late mRNAemia in the plasma could help identify episodes of active viral replication and could have the potential to shorten the duration of pre-emptive or prophylaxis strategies and aid the management of long-term infections, in particular when using drugs that inhibit CMV replication steps downstream of DNA polymerase, such as letermovir and maribavir [32]. Nevertheless, this tool is still undergoing investigation and validation.

**FIGURE 4:**
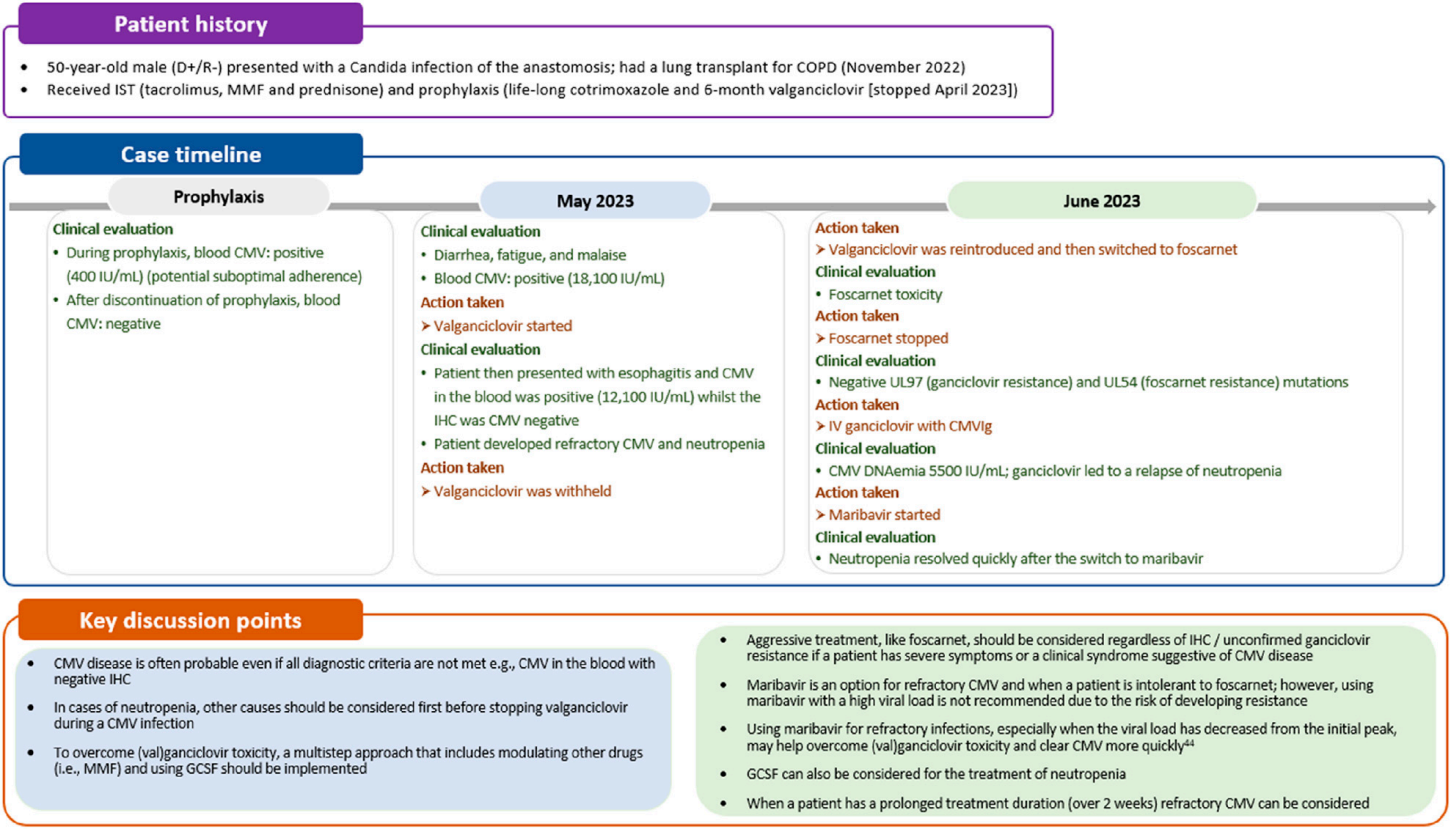
Case 2: Managing difficult to treat CMV disease. CMV, cytomegalovirus; COPD, chronic obstructive pulmonary disease; GCSF, granulocyte-colony stimulating factor; IHC, immunohistochemistry; IST, immunosuppressive therapy; MMF, mycophenolate mofetil.

The presence of CMV disease is possible even if all diagnostic criteria are not met and treatment should be initiated in such situations. Diagnostic criteria may vary depending on the organ involved, and relying solely on QNAT may not always be sufficient, particularly in GI tract-related CMV disease [33]. In the GI tract, end-organ disease can be evident (positive immunohistochemistry [IHC]) despite negative DNAemia [34]. Therefore, diagnosis of CMV GI disease requires the presence of upper and/or lower GI symptoms along with endoscopic evidence or laboratory confirmation of CMV infection [33]. Additionally, it is possible for biopsy samples to be taken from unaffected parts of the intestine in individuals with CMV GI disease. Confirmation of CMV pneumonia typically involves clinical signs and/or symptoms suggestive of pneumonia combined with laboratory confirmation of CMV in lung tissue [33]. In case 2, a lung transplant recipient exhibited CMV viremia but negative IHC results from an esophageal biopsy (Figure 4). Despite this negative IHC result for esophagitis, treatment for CMV disease was initiated in the patient. This decision was based on the fact that the patient had a clinical syndrome suggestive of CMV disease.

### Treatment of CMV Infection and Disease

First-line treatment of CMV infection or disease is oral valganciclovir or intravenous ganciclovir, with the latter often preferred in cases of life-threatening CMV, very high viral loads, or when oral absorption of medication is a concern [6]. Valganciclovir is a prodrug of ganciclovir, with both inhibiting viral DNA polymerases. In addition, these drugs can lead to varying degrees of bone marrow suppression and subsequently, neutropenia [35]. Oral valganciclovir has a longer half-life than intravenous ganciclovir meaning prolonged exposure (or slower clearance) of the drug and its metabolites [36]. Therefore, oral valganciclovir would be expected to be associated with a greater degree of neutropenia than intravenous ganciclovir, which was evident in study WV15376 (11% vs. 13%, respectively) [36, 37]. Higher incidences of neutropenia may be observed in patients with decreased kidney function, as this leads to reduced clearance of ganciclovir and a prolonged terminal half-life [36]. Neutropenia is one of the most common adverse event associated with valganciclovir, as was reported in case 2 (Figure 4) [38]. In cases of valganciclovir-induced neutropenia, dose adjustments or discontinuation of therapy, as well as management of other drugs, may be necessary, particularly if neutrophil counts fall below pre-defined thresholds or clinical symptoms of infection arise. Close monitoring of blood counts, particularly neutrophil levels, is crucial during valganciclovir therapy to manage neutropenia-related complications promptly.

Of note, as highlighted in case 2, neutropenia should not automatically prompt discontinuation of valganciclovir. Bone marrow suppression and neutropenia can also be caused by CMV infection itself [39], making it essential to assess whether neutropenia is a direct consequence of CMV replication. This may involve evaluating CMV viral load by DNAemia or other diagnostic tests to confirm active CMV infection. Furthermore, it is imperative to consider and investigate other potential underlying conditions or factors that could contribute to neutropenia. These may include medications commonly used in transplant recipients, such as immunosuppressive or anti-infective agents (e.g., mTOR inhibitors, mycophenolic acid, trimethoprim sulfamethoxazole). Additionally, concomitant viral infections (e.g., Epstein−Barr virus, Human Herpesvirus-8 [although relatively infrequent], and Parvovirus B19, adenovirus), hematologic disorders, or nutritional deficiencies need to be excluded. Once other potential causes are excluded, the initial consideration can be granulocyte-colony stimulating factor (GCSF) administration, followed by a possible switch to an alternative drug with a more acceptable safety profile (Figure 4) [35]. It is not recommended to use foscarnet or cidofovir to overcome valganciclovir toxicity, given their less acceptable safety profiles. Although it is preferred to only use these drugs as an alternative to valganciclovir in case of documented ganciclovir resistance, guidelines recommend considering foscarnet in refractory CMV cases with severe clinical symptoms or life-threatening disease [6]. Where available, maribavir can be considered for refractory or ganciclovir resistant infection in cases of intermediate viral loads, or as second step approach after an initial short course with foscarnet [6]. This approach is likely to minimize the toxicity of foscarnet and the risk of maribavir resistance that may occur when treating high viral loads.

Tailoring treatment approaches to each clinical scenario is essential for optimizing patient care. Impaired kidney function can lead to decreased drug clearance and increased drug concentrations, potentially increasing the risk of drug toxicity [40]. Therefore, in cases of kidney impairment, treatment doses should be adjusted according to kidney function to minimize adverse events, as highlighted in case 1 (Figure 2). However, despite these recommendations, data from kidney transplant recipients suggest that as many as one-third of patients may be receiving a dose of valganciclovir that is too high [41]. Immunosuppressive drugs, including corticosteroids, calcineurin inhibitors (e.g., tacrolimus and cyclosporine), and in particular antimetabolites (e.g., mycophenolate mofetil or mycophenolic acid), inhibit the immune response by suppressing the activity of immune cells, including T cells and natural killer cells [42], thus hindering immune surveillance and the ability to combat CMV infection. Switching or reducing immunosuppressive therapy should be considered as an adjunct to antiviral therapy to improve treatment outcomes, as demonstrated in case 1 (Figure 2).

Monitoring CMV viral load and complete blood count should be conducted on a weekly basis to guide the duration of therapy. Treatment should continue for a minimum of 2 weeks, until DNAemia falls below the detection threshold and signs and symptoms of CMV disease are resolved [6]. As introduced above, DNAemia may not accurately reflect CMV disease status in all clinical situations and longer courses of treatment may be needed, for example, in the treatment of tissue-invasive GI disease and pneumonitis in lung transplant recipients [6]. If a patient fails to respond after the recommended treatment duration with (val)ganciclovir, maribavir could be considered as an alternative option (Figure 4). However, as outlined earlier, we advise caution when using maribavir in patients with a high viral load due to the potential risk of selecting a resistant mutant [43]. Treatment failure may result from a resistant/refractory CMV infection or low adherence. Therapeutic drug monitoring, although not generally recommended in current guidelines, can be helpful in cases of suspected low adherence, or to ensure optimal drug levels in cases of kidney insufficiency, although a valganciclovir concentration clearly predictive of CMV clearance has not been determined [44]. If treatment fails in an adherent patient who meets the criteria for refractory disease, testing for resistant CMV should be considered.

### Treatment of Refractory/Resistant CMV Infection

Despite preventive strategies and well-established antiviral therapies, managing refractory/resistant CMV infection in patients undergoing SOT remains a significant challenge. Resistant/refractory CMV infection is defined as the failure to respond after 14 days of appropriate treatment [45]. Table 2 provides an overview of the definitions for refractory and resistant CMV infection. Ensuring appropriate dosing of antivirals is essential in the management of CMV infection, as suboptimal dosing can lead to an increased risk of treatment failure and resistance development [6].

**TABLE 2:** Summary of definitions of refractory and resistant CMV [46].

Term	Definition
Refractory CMV infection^a^	CMV virema (DNAemia or antigenemia) that either: 1. Has a >1 log_10_ increase in CMV DNA levels in the same blood compartment from the highest level previously measured in the same laboratory and/or with the same commercial assay) OR 2. Persists (≤1 log_10_ increase or decrease in CMV DNA levels) after ≥2 weeks of appropriate antiviral therapy.
Resistant CMV infection	Refractory infection with the presence of genetic mutations correlating to antiviral resistance, which leads to treatment failure

^a^ Refractory and probable refractory CMV infection are classified as one category.

In case of resistant CMV infection, mutations in the UL97 gene are most frequent, while UL54 mutations typically arise after prolonged pre-treatment and may lead to cross-resistance with cidofovir and foscarnet [45]. A laboratory study conducted in 2023 revealed CMV drug resistance in approximately 30% (n = 826/2750) of samples from transplant recipients sent for genotyping [47]. The most common resistance mutations in the UL97 gene were for ganciclovir and maribavir accounting for 27.64% and 9.96% of samples, respectively [47]. However, reported rates of CMV drug resistance may vary across publications. The annual reported incidence rate of ganciclovir resistance was less than 1% in 80% of transplant centers but reached up to 10% in some, according to the 2022 ESOT survey [26] and a recent trial of maribavir in patients with refractory or resistant CMV infection has reported a resistance rate in the region of 25% [43, 48]. Risk factors for resistant/refractory CMV infection include younger age, D+/R- serostatus, lung transplant, recurrent CMV infection, ongoing viral replication, prolonged antiviral treatment, subtherapeutic antiviral levels, high viral loads, and severe immunosuppression [45, 49]. Additionally, administering belatacept increases the risk of refractory CMV infection compared with other immunosuppressants. Belatacept was unable to sustain viral control relative to tacrolimus in high-risk recipients (n = 60) [50].

The latest international treatment recommendations for managing resistant CMV infection, as outlined in the 2025 guidelines, involves first reducing immunosuppression if feasible, followed by administering foscarnet, cidofovir, or high-dose ganciclovir depending on disease severity and genetic mutation type [6]. However, there is limited evidence supporting the use of high-dose ganciclovir. Additionally, older antivirals pose significant toxicity concerns, with ganciclovir linked to neutropenia, and foscarnet and cidofovir associated with a high risk of acute kidney injuries and increased mortality [51, 52].

Maribavir, an oral benzimidazole riboside antiviral, inhibits the CMV UL97 protein kinase involved in viral maturation and egress [53]. It was approved for the treatment of resistant/refractory CMV infection in the UK and Europe in 2022, with approval in the USA granted in 2021 [54, 55]. Maribavir is considered a valid alternative treatment for resistant/refractory CMV due to its more favorable safety profile [52]. A phase 2 study showed that ≥400 mg of maribavir twice daily achieved CMV clearance in SOT patients with resistant/refractory CMV [56]. The results from this study led to a large prospective phase 3 study in SOT and hematopoietic stem cell transplantation recipients (n = 352) with refractory CMV infection: after 8 weeks of therapy, maribavir showed greater CMV DNAemia clearance and fewer treatment discontinuations due to treatment-emergent adverse events compared with investigator-assigned therapy (valganciclovir/ganciclovir, cidofovir, or foscarnet) [52]. The viral response rate was 55.7%, compared with 23.9% in the investigator-assigned therapy group [52]. However, among the patients who achieved CMV clearance by Week 8 in the maribavir group, 66.4% of patients experienced a loss of response by Week 16 [52]. Alternative strategies, such as a longer treatment duration, should be evaluated, while also acknowledging the continued relevance of the conventional drugs, foscarnet and cidofovir, depending on the individual patient situation. However, it is important to note that from 6 weeks, maribavir can lead to CMV mutations and resistance in recurrent infections [57], and resistance to valganciclovir and maribavir in the same patient has been reported [58].

**FIGURE 5:**
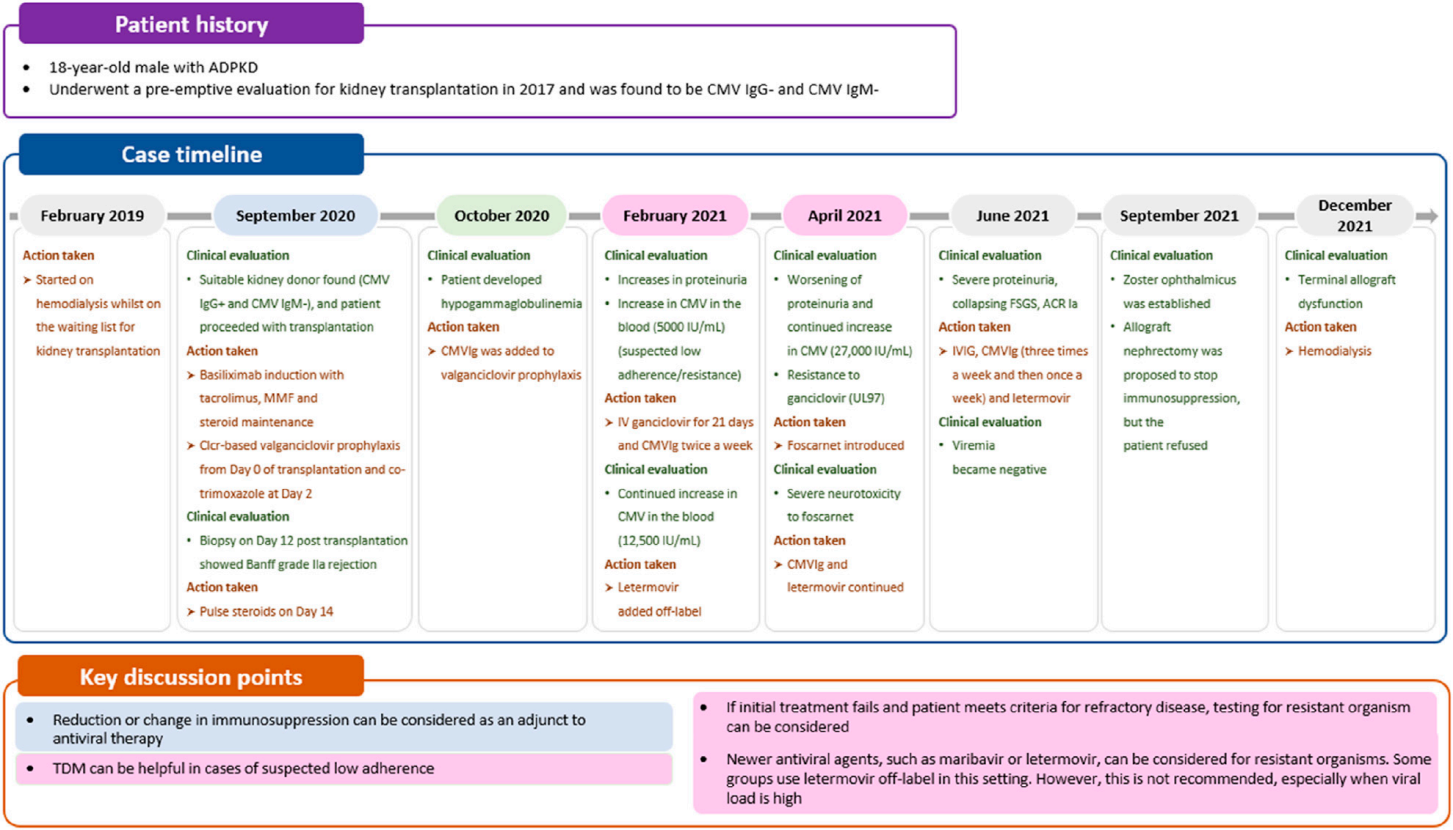
Case 3: Managing CMV resistance. ACR, acute cellular rejection; ADPKD, autosomal dominant polycystic kidney disease; CMV, cytomegalovirus; CMVIg, cytomegalovirus immunoglobulin; Clcr, creatinine clearance; FSGS, focal segmental glomerulosclerosis; IVIG, intravenous immunoglobulin; MMF, mycophenolate mofetil; TDM, therapeutic drug monitoring.

Letermovir disrupts the viral terminase complex (pUL56) and is currently approved for prophylaxis in patients undergoing hematopoietic stem cell transplantation or high-risk (D+/R-) kidney transplantation [59, 60]. Due to its more favorable safety profile and reduced risk of CMV resistance compared with valganciclovir, letermovir is sometimes used off-label for the treatment of resistant CMV, as observed in case 3 (Figure 5) [27]. However, there are concerns regarding the higher risk of resistant mutations, especially in patients with high viral loads, making letermovir potentially unsuitable in such patients [61].

Further “proof of concept” studies are needed to determine the role of letermovir in treating refractory infections and whether CMVIg can enhance T-cell response. In specific cases, combining CMVIg with antivirals may present a more viable approach. CMVIg can provide an additional mechanism of action by modulating the immune response through various mechanisms, including CMV neutralization, dendritic cell maturation modulation, decreased T-cell activation, and decreased cytokine production [62]. Although it is only licensed for prophylactic use, some clinicians use CMVIg off-label to support the treatment of resistant CMV infection. For example, in case 3, CMVIg was added to off-label letermovir treatment for a patient with hypogammaglobulinemia and ganciclovir-resistant CMV infection (Figure 5). Despite the potential benefits of CMVIg, there is limited evidence supporting its off-label use in the treatment of CMV infections [63, 64].

### The Role of Cell-Mediated Immunity (CMI) Monitoring in CMV Disease

The integration of CMI monitoring into the care pathway for CMV disease has the potential to revolutionize the management of CMV infection by offering a personalized approach to CMV management and enhanced care for individual patients [65]. CMI monitoring measures the production of interferon gamma, or other cytokines, produced by T cells in response to CMV antigens [66]. The level of CMI is commonly quantified using the ELISPOT or QuantiFERON-CMV assay [55]. Typically, a high CMI response indicates protection against CMV disease, whereas a low CMI response increases the risk of CMV reactivation or progression [66]. The impact of immunosuppressants on CMV-specific T-cell functionality varies [67], and by closely monitoring the immune response, preventive and curative strategies may be tailored appropriately [66, 68].

**FIGURE 6:**
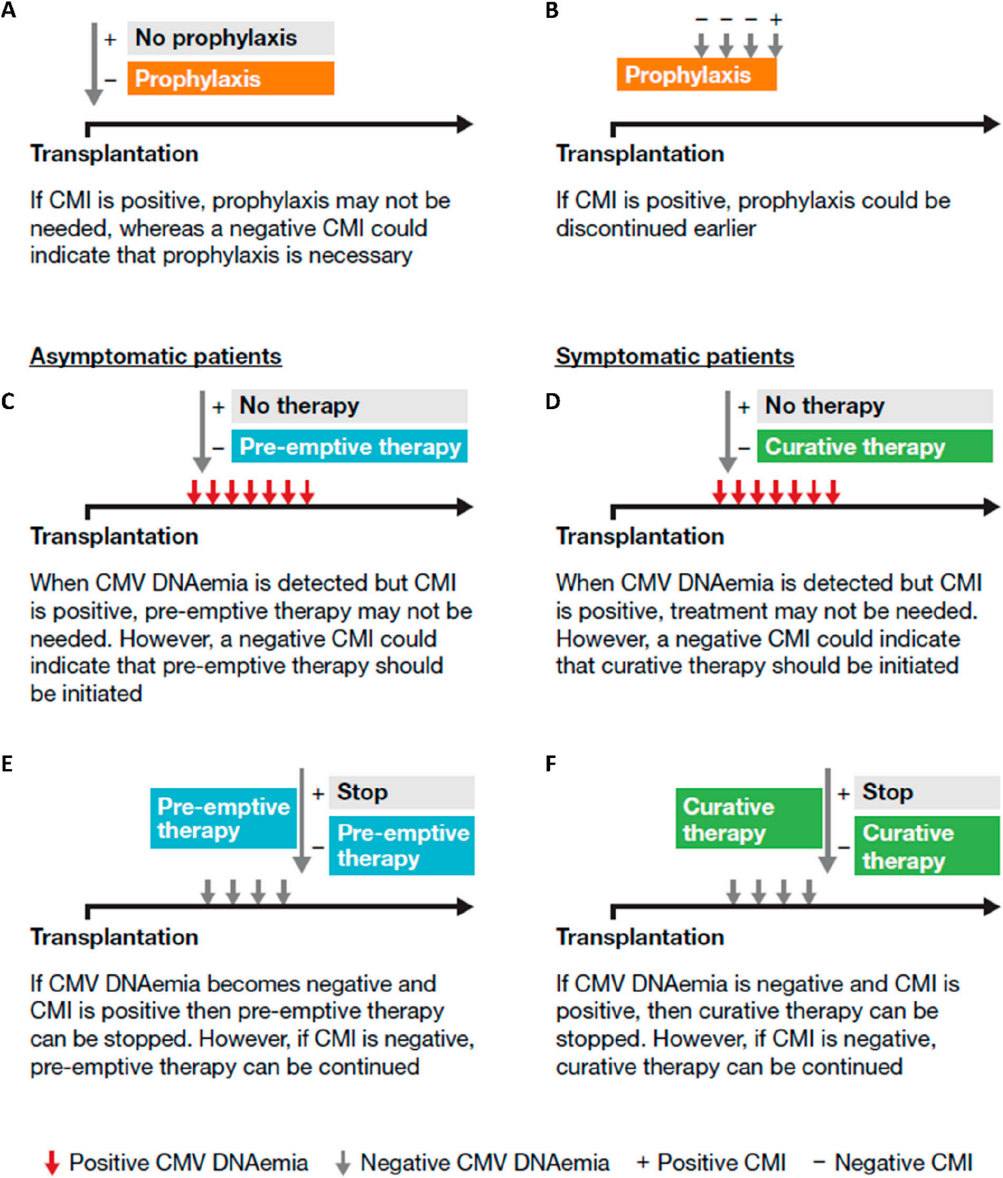
The potential use of CMI monitoring: **(A)** at transplantation; **(B)** during prophylaxis; at the onset of the infection in asymptomatic **(C)** and symptomatic patients **(D)**; **(E)** during pre-emptive therapy in asymptomatic patients; **(F)** at the end of the infection in symptomatic patients [69]. Based on Kaminski H, et al. Immunol Rev. 2020; 298:264–88.

CMI monitoring can be used as a decision-making tool at various stages of the patient journey (Figure 6) [65]. Unlike serology, which may misrepresent the risk of CMV infection in some patients, particularly D+/R+ patients, CMI is primarily driven by T cells and does not rely on B-cell antibody production [70]. CMI monitoring can be useful for stratifying the risk of CMV infection [31, 71, 72]. with the absence of pre-existing CMV-specific CMI in the recipient increasing the risk of CMV infection [31, 73]. In a prospective multicenter study in D+/R+ kidney recipients deemed to be at high risk for CMV based on pretransplant CMI significantly higher CMV infection rates were observed compared with those considered to be at lower risk, regardless of whether prophylaxis or pre-emptive protocols were followed [74]. However, with some immunosuppressive regimens additional comprehensive profiling of cytokine and chemokine responses may improve the performance of CMV-specific CMI [67, 68].

In case 1, CMI monitoring was used to assess the necessity of CMVIg treatment. An indeterminate QuantiFERON-CMV result, typically interpreted as negative, suggested low or no overall immunity, including against CMV, prompting the initiation of CMVIg. Additionally, CMI monitoring could have guided the decision on universal prophylaxis initiation or earlier initiation of valganciclovir (Figure 2). This approach allows low-risk patients to avoid unnecessary CMV preventive therapy and minimize their exposure to antivirals, consequently decreasing the associated risk of adverse effects [66].

If CMI is positive, prophylaxis could be discontinued earlier as was demonstrated in a recent study in D+/R- kidney and liver transplant recipients receiving prophylactic valganciclovir [75] and a similar study in CMV seropositive kidney transplant recipients receiving ATG [76]. Thus, CMI measurements could be used to tailor the duration of prophylaxis, particularly in high-risk patients, aiming to reduce the risk of toxicity [66]. However, although no difference in CMV disease and replication has been shown in some studies [76], others have been unable to establish non-inferiority of this CMI-guided approach on CMV infection [75]. In patients with low-level DNAemia, CMI testing serves as an adjunctive tool to guide the decision to initiate curative treatment in symptomatic patients, and to determine its optimal duration, or to guide pre-emptive therapy in asymptomatic patients [66]. Interestingly, patients with an indeterminate result appear to be over-immunosuppressed and have a greater risk of CMV reactivation than those with a negative result [66], as observed in case 1 where the patient, a high-risk individual post-rabbit ATG (rATG) administration, exhibited an indeterminate result (Figure 2).

Despite the promising findings from several studies, the integration of CMI monitoring into routine clinical practice still faces challenges due to certain limitations. These include the lack of a clearly defined protective threshold, timings, and accessibility. Where CMI is not available, clinicians can refer to alternative indicators of global immunity, such as lymphocyte count or hypogammaglobulinemia. Low levels of lymphocytes and immunoglobulins may indicate the need for additional interventions in patients at risk of CMV disease.

Before CMV-specific CMI monitoring can be integrated into routine clinical practice, several questions regarding immune-guided CMV management must be addressed. These include understanding why current CMI monitoring has a poor predictive value for D+/R- patients, explaining the reasons behind the occurrence of CMV infections in some R+ patients despite a positive QuantiFERON test result, and exploring the mechanisms enabling certain CMI−CMV patients to control CMV infection following curative treatment. Addressing these questions is essential for optimizing the utility of CMI monitoring in personalized CMV management strategies.

## Conclusion

The management of CMV presents complex challenges, underscoring the necessity to standardize CMV management through an evidence-based approach. The workshop highlighted the need for further close collaboration between experts in the field to continue optimizing CMV management. Newer antivirals, such as maribavir, could reduce antiviral-associated toxicity in resistant/refractory CMV infections, but limitations remain. CMI is increasingly being employed to make key decisions throughout the patient’s treatment journey however, more information is required before CMI becomes a part of routine practice. ESOT will continue to try to streamline and optimize the management of CMV infection and disease in this challenging population.

## References

[1] Al Mana H Yassine HM Younes NN Al-Mohannadi A Al-Sadeq DW Alhababi D The Current Status of Cytomegalovirus (CMV) Prevalence in the MENA Region: A Systematic Review. Pathogens (2019) 8:213. 10.3390/pathogens8040213 31683687 PMC6963600

[2] Zuhair M Smit GSA Wallis G Jabbar F Smith C Devleesschauwer B Estimation of the Worldwide Seroprevalence of Cytomegalovirus: A Systematic Review and Meta-Analysis. Rev Med Virol (2019) 29:e2034. 10.1002/rmv.2034 30706584

[3] Antona D Lepoutre A Fonteneau L Baudon C Halftermeyer-Zhou F Le Strat Y Seroprevalence of Cytomegalovirus Infection in France in 2010. Epidemiol Infect (2017) 145:1471–8. 10.1017/s0950268817000103 28166842 PMC9203352

[4] Vilibic-Cavlek T Kolaric B Beader N Vrtar I Tabain I Mlinaric-Galinovic G. Seroepidemiology of Cytomegalovirus Infections in Croatia. Wien Klin Wochenschr (2017) 129:129–35. 10.1007/s00508-016-1069-7 27599701

[5] Azevedo LS Pierrotti LC Abdala E Costa SF Strabelli TM Campos SV Cytomegalovirus Infection in Transplant Recipients. Clinics (Sao Paulo) (2015) 70:515–23. 10.6061/clinics/2015(07)09 26222822 PMC4496754

[6] Kotton CN Kumar D Manuel O Chou S Hayden RT Danziger-Isakov L The Fourth International Consensus Guidelines on the Management of Cytomegalovirus in Solid-Organ Transplantation. Transplantation (2025) 109(Apr 9):1066–110. 10.1097/TP.0000000000005374 40200403 PMC12180710

[7] Reischig T Kacer M Hruba P Jindra P Hes O Lysak D The Impact of Viral Load and Time to Onset of Cytomegalovirus Replication on Long-Term Graft Survival After Kidney Transplantation. Antivir Ther (2017) 22:503–13. 10.3851/IMP3129 28091392

[8] Leeaphorn N Garg N Thamcharoen N Khankin EV Cardarelli F Pavlakis M. Cytomegalovirus Mismatch Still Negatively Affects Patient and Graft Survival in the Era of Routine Prophylactic and Preemptive Therapy: A Paired Kidney Analysis. Am J Transpl (2019) 19:573–84. 10.1111/ajt.15183 30431703

[9] Tavakol M Ashimine S Braun H Cleveland H Laszik Z Roberts J. P8.146: Early and Late-Onset Cytomegalovirus Infection Following Universal Prophylaxis in Kidney Transplant Recipients: Risk Factors and Outcome. Transplantation (2022) 106:S628. 10.1097/01.tp.0000888968.56630.fb

[10] Navarro D San-Juan R Manuel O Giménez E Fernández-Ruiz M Hirsch HH Cytomegalovirus Infection Management in Solid Organ Transplant Recipients Across European Centers in the Time of Molecular Diagnostics: An ESGICH Survey. Transpl Infect Dis (2017) 19:e12773. 10.1111/tid.12773 28859257

[11] Engelmann C Sterneck M Weiss KH Templin S Zopf S Denk G Prevention and Management of CMV Infections After Liver Transplantation: Current Practice in German Transplant Centers. J Clin Med (2020) 9:2352. 10.3390/jcm9082352 32717978 PMC7465768

[12] Kotton CN. CMV: Prevention, Diagnosis and Therapy. Am J Transpl (2013) 13:24–40. 10.1111/ajt.12006 23347212

[13] Griffiths P Reeves M. Pathogenesis of Human Cytomegalovirus in the Immunocompromised Host. Nat Rev Microbiol (2021) 19:759–73. 10.1038/s41579-021-00582-z 34168328 PMC8223196

[14] Hill RB Rowlands DT Rifkind D. Infectious Pulmonary Disease in Patients Receiving Immunosuppressive Therapy for Organ Transplantation. N Engl J Med (1964) 271:1021–7. 10.1056/nejm196411122712001 14195753

[15] Grattan MT Moreno-Cabral CE Starnes VA Oyer PE Stinson EB Shumway NE. Cytomegalovirus Infection Is Associated with Cardiac Allograft Rejection and Atherosclerosis. JAMA (1989) 261:3561–6. 10.1001/jama.1989.03420240075030 2542633

[16] Toupance O Bouedjoro-Camus MC Carquin J Novella JL Lavaud S Wynckel A Cytomegalovirus-Related Disease and Risk of Acute Rejection in Renal Transplant Recipients: A Cohort Study with Case-Control Analyses. Transpl Int (2000) 13:413–9. 10.1007/s001470050723 11140239

[17] Stern M Hirsch H Cusini A van Delden C Manuel O Meylan P Cytomegalovirus Serology and Replication Remain Associated with Solid Organ Graft Rejection and Graft Loss in the Era of Prophylactic Treatment. Transplantation (2014) 98:1013–8. 10.1097/tp.0000000000000160 24837540

[18] Kobashigawa J Ross H Bara C Delgado JF Dengler T Lehmkuhl HB Everolimus Is Associated with a Reduced Incidence of Cytomegalovirus Infection Following De Novo Cardiac Transplantation. Transpl Infect Dis (2013) 15:150–62. 10.1111/tid.12007 23013440

[19] British Transplant Society. UK Guideline on Prevention and Management of Cytomegalovirus (CMV) Infection and Disease Following Solid Organ Transplantation (2021). Available online at: https://bts.org.uk/uk-guideline-on-prevention-and-management-of-cytomegalovirus-cmv-infection-and-disease-following-solid-organ-transplantation/ (Accessed May 30, 2025).

[20] Manuel O Avery RK. Update on Cytomegalovirus in Transplant Recipients: New Agents, Prophylaxis, and Cell-Mediated Immunity. Curr Opin Infect Dis (2021) 34:307–13. 10.1097/qco.0000000000000746 34074879

[21] Singh N Winston DJ Razonable RRR Lyon GM Silveira SFP Wagener M Effect of Preemptive Therapy vs Antiviral Prophylaxis on Cytomegalovirus Disease in Seronegative Liver Transplant Recipients with Seropositive Donors: A Randomized Clinical Trial. JAMA (2020) 323:1378–87. 10.1001/jama.2020.3138 32286644 PMC7157180

[22] Reischig T Vlas T Kacer M Pivovarcikova K Lysak D Nemcova J A Randomized Trial of Valganciclovir Prophylaxis Versus Preemptive Therapy in Kidney Transplant Recipients. J Am Soc Nephrol (2023) 34:920–34. 10.1681/ASN.0000000000000090 36749127 PMC10125645

[23] Raval AD Kistler K Tang Y Murata Y Snydman DR. Antiviral Treatment Approaches for Cytomegalovirus Prevention in Kidney Transplant Recipients: A Systematic Review of Randomized Controlled Trials. Transpl Rev (Orlando) (2021) 35:100587. 10.1016/j.trre.2020.100587 33190040

[24] Owers DS Webster AC Strippoli GF Kable K Hodson EM. Pre-Emptive Treatment for Cytomegalovirus Viraemia to Prevent Cytomegalovirus Disease in Solid Organ Transplant Recipients. Cochrane Database Syst Rev (2013) 2013:Cd005133. 10.1002/14651858.CD005133.pub3 23450558 PMC6823220

[25] Styczyński J. Prophylaxis vs Preemptive Therapy in Prevention of CMV Infection: New Insight on Prophylactic Strategy After Allogeneic Hematopoietic Cell Transplantation. Acta Haematol Pol (2020) 51:17–23. 10.2478/ahp-2020-0005

[26] Grossi PA Kamar N Saliba F Baldanti F Aguado JM Gottlieb J Cytomegalovirus Management in Solid Organ Transplant Recipients: A pre-COVID-19 Survey from the Working Group of the European Society for Organ Transplantation. Transpl Int (2022) 35:10332. 10.3389/ti.2022.10332 35812158 PMC9257585

[27] Limaye AP Budde K Humar A Vincenti F Kuypers DRJ Carroll RP Letermovir vs Valganciclovir for Prophylaxis of Cytomegalovirus in High-Risk Kidney Transplant Recipients: A Randomized Clinical Trial. JAMA (2023) 330:33–42. 10.1001/jama.2023.9106 37279999 PMC10245286

[28] Barten MJ Baldanti F Staus A Hüber CM Glynou K Zuckermann A. Effectiveness of Prophylactic Human Cytomegalovirus Hyperimmunoglobulin in Preventing Cytomegalovirus Infection Following Transplantation: A Systematic Review and Meta-Analysis. Life (Basel) (2022) 12:361. 10.3390/life12030361 35330112 PMC8955988

[29] Solidoro P Patrucco F Libertucci D Verri G Sidoti F Curtoni A Tailored Combined Cytomegalovirus Management in Lung Transplantation: A Retrospective Analysis. Ther Adv Respir Dis (2019) 13:1753466619878555. 10.1177/1753466619878555 31566097 PMC6769221

[30] Potena L Holweg CT Chin C Luikart H Weisshaar D Narasimhan B Acute Rejection and Cardiac Allograft Vascular Disease Is Reduced by Suppression of Subclinical Cytomegalovirus Infection. Transplantation (2006) 82:398–405. 10.1097/01.tp.0000229039.87735.76 16906040

[31] Lee H Oh EJ. Laboratory Diagnostic Testing for Cytomegalovirus Infection in Solid Organ Transplant Patients. Korean J Transpl (2022) 36:15–28. 10.4285/kjt.22.0001 35769434 PMC9235525

[32] Piccirilli G Lanna F Gabrielli L Motta V Franceschiello M Cantiani A CMV-RNAemia as New Marker of Active Viral Replication in Transplant Recipients. J Clin Microbiol (2024) 62:e0163023. 10.1128/jcm.01630-23 38534109 PMC11078005

[33] Ljungman P Boeckh M Hirsch HH Josephson F Lundgren J Nichols G Definitions of Cytomegalovirus Infection and Disease in Transplant Patients for Use in Clinical Trials. Clin Infect Dis (2017) 64:87–91. 10.1093/cid/ciw668 27682069

[34] Suárez-Lledó M Marcos M Cuatrecasas M Bombi JA Fernández-Avilés F Magnano L Quantitative PCR Is Faster, More Objective, and More Reliable than Immunohistochemistry for the Diagnosis of Cytomegalovirus Gastrointestinal Disease in Allogeneic Stem Cell Transplantation. Biol Blood Marrow Transpl (2019) 25:2281–6. 10.1016/j.bbmt.2019.07.016 31325586

[35] Kalil AC Freifeld AG Lyden ER Stoner JA. Valganciclovir for Cytomegalovirus Prevention in Solid Organ Transplant Patients: An Evidence-Based Reassessment of Safety and Efficacy. PLoS One (2009) 4:e5512. 10.1371/journal.pone.0005512 19436751 PMC2677673

[36] Roche. VALCYTE (Valganciclovir Hydrochloride Tablets) (2001). Available online at: https://www.accessdata.fda.gov/drugsatfda_docs/label/2001/21304lbl.pdf (Accessed May 30, 2025)

[37] Genentech. Highlights of Prescribing Information (2021). Available online at: https://www.gene.com/download/pdf/valcyte_prescribing.pdf (Accessed May 30, 2025).

[38] Martín-Gandul C Pérez-Romero P González-Roncero FM Berdaguer S Gómez MA Lage E Clinical Impact of Neutropenia Related with the Preemptive Therapy of CMV Infection in Solid Organ Transplant Recipients. J Infect (2014) 69:500–6. 10.1016/j.jinf.2014.07.001 25037022

[39] Salzberger B Bowden RA Hackman RC Davis C Boeckh M. Neutropenia in Allogeneic Marrow Transplant Recipients Receiving Ganciclovir for Prevention of Cytomegalovirus Disease: Risk Factors and Outcome. Blood (1997) 90:2502–8. 10.1182/blood.v90.6.2502 9310503

[40] MacIntyre IM. Prescribing Medicines for Patients with Renal Impairment. Medicine (2024) 52:31–5. 10.1016/j.mpmed.2023.10.009

[41] Hammer N Hoessly L Haidar F Hirzel C de Seigneux S van Delden C Pitfalls in Valganciclovir Prophylaxis Dose Adjustment Based on Renal Function in Kidney Transplant Recipients. Transpl Int (2024) 37:12712. 10.3389/ti.2024.12712 38784442 PMC11112565

[42] Zaza G Leventhal J Signorini L Gambaro G Cravedi P. Effects of Antirejection Drugs on Innate Immune Cells After Kidney Transplantation. Front Immunol (2019) 10:2978. 10.3389/fimmu.2019.02978 31921213 PMC6930910

[43] Chou S Alain S Cervera C Chemaly RF Kotton CN Lundgren J Drug Resistance Assessed in a Phase 3 Clinical Trial of Maribavir Therapy for Refractory or Resistant Cytomegalovirus Infection in Transplant Recipients. J Infect Dis (2024) 229:413–21. 10.1093/infdis/jiad293 37506264 PMC10873177

[44] Gatti M Rinaldi M Potena L Salvaterra E Morelli MC Giannella M Does Therapeutic Drug Monitoring (TDM) of Trough Concentrations Suffice for Optimizing Preemptive Therapy with Ganciclovir of Cytomegalovirus Infections in Non-Renal Solid Organ Transplant Recipients? Transpl Infect Dis (2023) 25:e14107. 10.1111/tid.14107 37515787

[45] Walti CS Khanna N Avery RK Helanterä I. New Treatment Options for refractory/resistant CMV Infection. Transpl Int (2023) 36:11785. 10.3389/ti.2023.11785 37901297 PMC10600348

[46] Ljungman P Chemaly RF Khawaya F Alain S Avery R Badshah C Consensus Definitions of Cytomegalovirus (CMV) Infection and Disease in Transplant Patients Including Resistant and Refractory CMV for Use in Clinical Trials: 2024 Update from the Transplant Associated Virus Infections Forum. Clin Infect Dis (2024) 79:787–94. 10.1093/cid/ciae321 39041385 PMC11426271

[47] Kleiboeker SB. Prevalence of Cytomegalovirus Antiviral Drug Resistance in Transplant Recipients. Antivir Res (2023) 215:105623. 10.1016/j.antiviral.2023.105623 37150409

[48] Chou S Winston DJ Avery RK Cordonnier C Duarte RF Haider S Comparative Emergence of Maribavir and Ganciclovir Resistance in a Randomized Phase 3 Clinical Trial for Treatment of Cytomegalovirus Infection. J Infect Dis (2025) 231:e470–e477. 10.1093/infdis/jiae469 39302855 PMC11911792

[49] Tamzali Y Pourcher V Azoyan L Ouali N Barrou B Conti F Factors Associated with Genotypic Resistance and Outcome Among Solid Organ Transplant Recipients with Refractory Cytomegalovirus Infection. Transpl Int (2023) 36:11295. 10.3389/ti.2023.11295 37398559 PMC10307959

[50] Magua W Johnson AC Karadkhele GM Badell IR Vasanth P Mehta AK Impact of Belatacept and Tacrolimus on Cytomegalovirus Viral Load Control and Relapse in Moderate and High-Risk Cytomegalovirus Serostatus Kidney Transplant Recipients. Transpl Infect Dis (2022) 24:e13983. 10.1111/tid.13983 36321801 PMC10078597

[51] Joyce EL Kane-Gill SL Fuhrman DY Kellum JA. Drug-Associated Acute Kidney Injury: Who's at Risk? Pediatr Nephrol (2017) 32:59–69. 10.1007/s00467-016-3446-x 27338726 PMC5826624

[52] Avery RK Alain S Alexander BD Blumberg EA Chemaly RF Cordonnier C Maribavir for Refractory Cytomegalovirus Infections with or Without Resistance Post-Transplant: Results from a Phase 3 Randomized Clinical Trial. Clin Infect Dis (2022) 75:690–701. 10.1093/cid/ciab988 34864943 PMC9464078

[53] Sun K Fournier M Sundberg AK Song IH. Maribavir: Mechanism of Action, Clinical, and Translational Science. Clin Transl Sci (2023) 17:e13696. 10.1111/cts.13696 38071422 PMC10801391

[54] FirstWordPharma. Press Release: NICE Recommends LIVTENCITY (Maribavir) for the Treatment of Adults with Post-Transplant Cytomegalovirus (CMV) Refractory (With or Without Resistance) to Prior Therapies (2022). Available online at: https://firstwordpharma.com/story/5684722 (Accessed May 30, 2025).

[55] EMA. Annex I Summary of Product Characteristics (2022). Available online at: https://www.ema.europa.eu/en/documents/product-information/livtencity-epar-product-information_en.pdf (Accessed May 30, 2025).

[56] Papanicolaou GA Silveira FP Langston AA Pereira MR Avery RK Uknis M Maribavir for Refractory or Resistant Cytomegalovirus Infections in Hematopoietic-Cell or Solid-Organ Transplant Recipients: A Randomized, Dose-Ranging, Double-Blind, Phase 2 Study. Clin Infect Dis (2019) 68:1255–64. 10.1093/cid/ciy706 30329038 PMC6451997

[57] Chou S Song K Wu J Bo T Crumpacker C. Drug Resistance Mutations and Associated Phenotypes Detected in Clinical Trials of Maribavir for Treatment of Cytomegalovirus Infection. J Infect Dis (2022) 226:576–84. 10.1093/infdis/jiaa462 32726419 PMC9441206

[58] Pearce H Montgomery EK Sheerin N Ellam H. A Novel Case of CMV Resistance to Valganciclovir and Maribavir in a Renal Transplant Patient. Transpl Int (2024) 37:11985. 10.3389/ti.2024.11985 38314399 PMC10834638

[59] Verghese PS Schleiss MR. Letermovir Treatment of Human Cytomegalovirus Infection Antiinfective Agent. Drugs Future (2013) 38:291–8. 10.1358/dof.2013.038.05.1946425 24163496 PMC3807861

[60] EMA. Prevymis (Letermovir) (2024). Available online at: https://www.ema.europa.eu/en/documents/overview/prevymis-epar-summary-public_en.pdf (Accessed May 30, 2025).

[61] Cochran WV Dioverti MV Langlee J Barker LN Shedeck A Toman LP Approaches and Challenges in the Current Management of Cytomegalovirus in Transplant Recipients: Highlighting the Role of Advanced Practice Providers (Nurse Practitioners and Physician Assistants). Ann Transpl (2024) 29:e941185. 10.12659/aot.941185 38650316 PMC11055468

[62] Kotton CN Torre-Cisneros J Aguado JM Alain S Baldanti F Baumann G Cytomegalovirus in the Transplant Setting: Where Are We now and what Happens Next? A Report from the International CMV Symposium 2021. Transpl Infect Dis (2022) 24:e13977. 10.1111/tid.13977 36271650 PMC10078482

[63] Schulz U Solidoro P Müller V Szabo A Gottlieb J Wilkens H CMV Immunoglobulins for the Treatment of CMV Infections in Thoracic Transplant Recipients. Transplantation (2016) 100:S5–10. 10.1097/tp.0000000000001097 26900992 PMC4764017

[64] Whittaker J Martinez A Dains JE. Role of Preemptive Cytomegalovirus Hyperimmunoglobulin in Cytomegalovirus Viremia Following Stem Cell Transplant: An Integrative Review. J Adv Pract Oncol (2023) 14:620–30. 10.6004/jadpro.2023.14.7.6 38196668 PMC10715284

[65] Bestard O Kaminski H Couzi L Fernández-Ruiz M Manuel O. Cytomegalovirus Cell-Mediated Immunity: Ready for Routine Use? Transpl Int (2023) 36:11963. 10.3389/ti.2023.11963 38020746 PMC10661902

[66] Hall VG Humar A Kumar D. Utility of Cytomegalovirus Cell-Mediated Immunity Assays in Solid Organ Transplantation. J Clin Microbiol (2022) 60:e0171621. 10.1128/jcm.01716-21 35543099 PMC9383112

[67] Krueger MB Bonifacius A Dragon AC Santamorena MM Nashan B Taubert R *In Vitro* Profiling of Commonly Used Post-Transplant Immunosuppressants Reveals Distinct Impact on Antiviral T-cell Immunity Towards CMV. Transpl Int (2024) 37:12720. 10.3389/ti.2024.12720 38655204 PMC11035762

[68] Fernández-Ruiz M Parra P Ruiz-Merlo T Redondo N Rodríguez-Goncer I Andrés A Cytokine and Chemokine Secretome and Risk of CMV Infection Following Discontinuation of Valganciclovir Prophylaxis. Transpl Int (2023) 36:10979. 10.3389/ti.2023.10979 36776902 PMC9908579

[69] Kaminski H Marsères G Cosentino A Guerville F Pitard V Fournié JJ Understanding Human Γδ T Cell Biology Toward a Better Management of Cytomegalovirus Infection. Immunol Rev (2020) 298:264–88. 10.1111/imr.12922 33091199

[70] Marshall JS Warrington R Watson W Kim HL. An Introduction to Immunology and Immunopathology. Allergy Asthma Clin Immunol (2018) 14:49. 10.1186/s13223-018-0278-1 30263032 PMC6156898

[71] Solidoro P Curtoni A Patrucco F Russo E Sidoti F Piccinini G Quantiferon® Monitor Test as a Potential Tool for Stratifying Patients by Infection Risk and Tailoring Follow-Up Care in Lung Transplant Recipients: A Single-Center Retrospective Experience. Microorganisms (2025) 13:316. 10.3390/microorganisms13020316 40005684 PMC11858317

[72] Namsiripongpun W Kantachuvesiri S Bruminhent J. Utility of the Interferon-Gamma Enzyme-Linked Immunosorbent Spot Assay to Predict Risk of Cytomegalovirus Infection in Kidney Transplant Recipients. Transpl Int (2024) 36:11527. 10.3389/ti.2023.11527 38249787 PMC10796607

[73] Chiereghin A Potena L Borgese L Gibertoni D Squarzoni D Turello G Monitoring of Cytomegalovirus (CMV)-Specific Cell-Mediated Immunity in Heart Transplant Recipients: Clinical Utility of the QuantiFERON-CMV Assay for Management of Posttransplant CMV Infection. J Clin Microbiol (2018) 56:e01040-17. 10.1128/JCM.01040-17 29305542 PMC5869821

[74] Jarque M Crespo E Melilli E Gutiérrez A Moreso F Guirado L Cellular Immunity to Predict the Risk of Cytomegalovirus Infection in Kidney Transplantation: A Prospective, Interventional, Multicenter Clinical Trial. Clin Infect Dis (2020) 71:2375–85. 10.1093/cid/ciz1209 32076718

[75] Manuel O Laager M Hirzel C Neofytos D Walti LN Hoenger G Immune Monitoring-Guided Versus Fixed Duration of Antiviral Prophylaxis Against Cytomegalovirus in Solid-Organ Transplant Recipients: A Multicenter, Randomized Clinical Trial. Clin Infect Dis (2024) 78:312–23. 10.1093/cid/ciad575 37738676 PMC10874264

[76] Páez-Vega A Gutiérrez-Gutiérrez B Agüera ML Facundo C Redondo-Pachón D Suñer M Immunoguided Discontinuation of Prophylaxis for Cytomegalovirus Disease in Kidney Transplant Recipients Treated with Antithymocyte Globulin: A Randomized Clinical Trial. Clin Infect Dis (2022) 74:757–65. 10.1093/cid/ciab574 34228099

